# Characterization and Structure Prediction of Partial Length Protein Sequences of *pcoA, pcoR* and *chrB* Genes from Heavy Metal Resistant Bacteria from the Klip River, South Africa

**DOI:** 10.3390/ijms16047352

**Published:** 2015-04-01

**Authors:** Patience Chihomvu, Peter Stegmann, Michael Pillay

**Affiliations:** Department of Biotechnology, Vaal University of Technology, Private Bag X021, Vanderbijlpark 1900, South Africa; E-Mails: stegmannfam@mweb.co (P.S.); mpillay@vut.ac.za (M.P.)

**Keywords:** pcoA, pcoR, chrB, Klip River, I-TASSER, homology modeling, partial protein

## Abstract

The Klip River has suffered from severe anthropogenic effects from industrial activities such as mining. Long-term exposure to heavy metal pollution has led to the development of heavy metal resistant strains of *Pseudomonas* sp. KR23, *Lysinibacillus* sp. KR25, and *E. coli* KR29. The objectives of this study were to characterize the genetics of copper and chromate resistance of the isolates. Copper and chromate resistance determinants were cloned and sequenced. Open reading frames (ORFs) related to the genes CopA and CopR were identified in *E. coli* KR29, PcoA in *Lysinibacillus* sp. KR25 and none related to chromate resistance were detected. The 3D-models predicted by I-TASSER disclose that the PcoA proteins consist of β-sheets, which form a part of the cupredoxin domain of the CopA copper resistance family of genes. The model for PcoR_29 revealed the presence of a helix turn helix; this forms part of a DNA binding protein, which is part of a heavy metal transcriptional regulator. The bacterial strains were cured using ethidium bromide. The genes encoding for heavy metal resistance and antibiotic resistance were found to be located on the chromosome for both *Pseudomonas* sp. (KR23) and *E. coli* (KR29). For *Lysinibacillus* (KR25) the heavy metal resistance determinants are suspected to be located on a mobile genetic element, which was not detected using gel electrophoresis.

## 1. Introduction

Heavy metal contamination of surface waters has a direct impact on the environment and public health. Microorganisms inhabiting such environments rapidly adapt and are sensitive to low concentrations of heavy metals. Therefore they can be used as bio-indicators to detect heavy metal pollution in the environment [1]. Evidence from previous studies [2,3] showed that the Klip River System is contaminated with heavy metals from various sources especially mine tailings. The Klip River harbors a number of heavy metal resistant microorganisms that are resistant to different levels of chromate and copper [3]. Bacteria have developed coping mechanisms that enable them to survive harsh environments with toxic levels of drugs and metals. These mechanisms include intrinsic biochemical, structural, and physiological properties and genetic adaptations [4].

Although copper is an essential element for microorganisms, excess copper in the environment is toxic to them [5]. Consequently, bacteria have developed copper resistance mechanisms. Copper resistance is usually conferred by genes found in plasmids of bacteria, namely *E. coli* [6,7] and *Pseudomonas* spp. [8]. There are basically four structural genes (*pcoA, pcoB, pcoC* and *pcoD*) involved in copper resistance in *E.coli.* PcoA is a multicopper oxidase, which is able to oxidise PcoC-bound copper (I) to its less toxic form copper (II) [9,10]. The structural genes present in *E. coli* are homologous to those found in *Pseudomonas* spp. (copA, copB, copC and copD) [11]. Moreover, *E. coli* and *Pseudomonas* both have a paired regulatory gene with a membrane bound copper (II) sensor. This is a product of the *pcoS* and *copS* genes that function together with the *pcoR* and *copR* genes, which are DNA binding repressor proteins [6,7]. Therefore, the copper resistance mechanisms present in *E. coli* are more or less similar to those that exist in *Pseudomonas* spp. The complete pcoABCDRSE operon was identified in *Enterobacter* sp. isolated from oil and petrol contaminated sites [12].

Chromate resistance is related to the presence of chromosomal or plasmid encoded genes [13]. For example, the most common plasmid pMOL28 of *Cuprividus metallidurans* harbors the *chrBAC* genes responsible for chromium resistance [14]. The chromate efflux system is encoded by the *chrA* gene. This gene is responsible for chromate resistance in *Shewanella* spp. [15]. The chrA protein belongs to the CHR superfamily of transporters [16]. The *chrB* gene encodes a membrane bound protein necessary for the regulation of chromate resistance [17]. The *ChrC* gene encodes a protein almost similar to iron-containing superoxide dismutase while the *chrE* gene encodes a gene product that is a rhodanese type enzyme and has been detected in *Orthrobacterium tritici* 5bvI1 [17]; *chrF* most probably encodes a repressor for chromate-dependent induction [16]. The latter gene has also been detected in *Orthrobacterium tritici* [17]. Genes responsible for chromate resistance have also been found in the following microorganisms: *Pseudomanas* spp. [18], *Streptococcus lactis* [19], *Arthrobacter* [20]. The *chrB* gene was detected in several microorganisms, namely, *Pseudomonas putida*, *Bacillus licheniformis*, *Bacillus cereus*, *Brevibacillus laterosporus*, *Trachelophylum* sp. *Peranema*, and *Adispica* sp. [21]. A putative chromate transport operon *chrIA1* and *chrA* genes, which encode putative chromate transporters were identified by genomic sequence analysis in *Orthrobacterium tritici* 5bvI1 [17]. *Orthrobacterium tritici* 5bvI1 was also found to be resistant to very high levels of chromate and the expression of an inducible chromate-resistant gene was detected on the mobile elements (TnOtChr), which possesses the genes *chrB*, *chrA*, *chrC* and *chrF* [17].

Protein structure is crucial in understanding its function. In the absence of experimentally determined protein structures, computational methods can be used to accurately predict protein structures from RNA sequences [22]. Functional proteins basically have a relatively stable structure. Given an arbitrary protein sequence, it would be informative to accurately predict the probability that the partial protein sequence represents a foldable protein sequence. This information would be valuable in genome annotation by supporting or rejecting hypothetical proteins stemming from unfamiliar regions of DNA [23]. Having an accurately predicted structure of an uncharacterized partial protein sequence is likely to provide information about its function that a sequence alone cannot provide [24,25].

The presence of plasmids in bacteria can have a major impact on their metabolism. However, the plasmid genes could be removed by heterocyclic compounds that bind to the plasmid DNA in a process known as plasmid curing. Plasmid curing is the loss of plasmids from a bacterial cell leading to the loss of specific phenotypes, such as antibiotic resistance [26]. Plasmid removal can reverse heavy metal and antibiotics resistance. Elimination of plasmids from bacteria can be carried out on strains grown as pure or mixed cultures in the presence of sub-inhibitory concentrations of non-mutagenic heterocyclic compounds [27].

The present work was aimed at characterizing the copper and chromate resistance genes that exist in heavy metal resistant bacteria isolated from the Klip River by using molecular and bioinformatics methods. In this study, growth curve characteristics were determined. The presence of copper and chromate resistance genes were detected and sequenced. The nucleotide sequences were translated to protein sequences, 3D-protein structures and functions were predicted using bioinformatics tools. The isolates were further characterized to determine the location of resistance determinants.

## 2. Results and Discussion

### 2.1. Growth Curve Analysis

**Figure 1:**
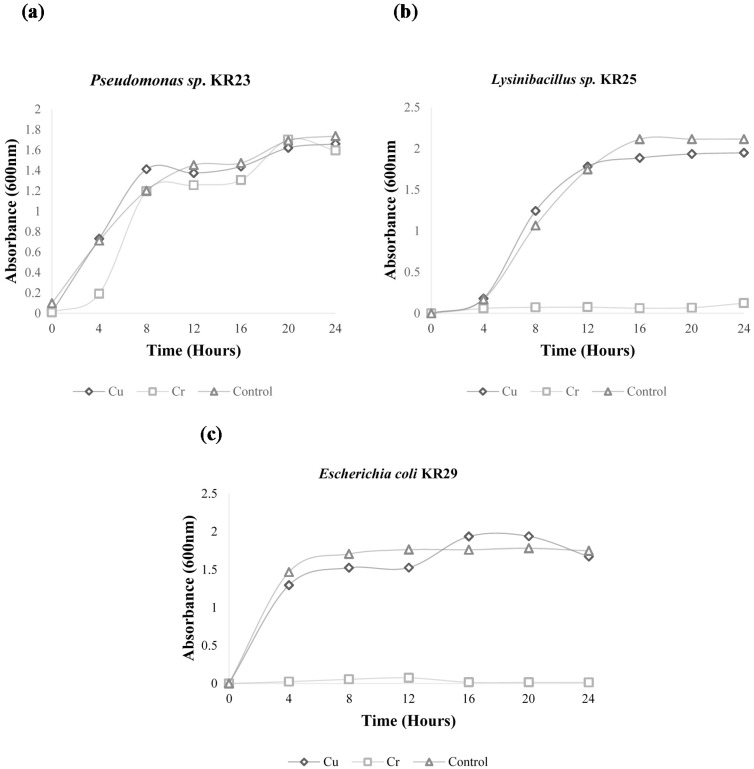
Growth curves of (**a**) *Pseudomonas* sp. KR23; (**b**) *Lyisnibacillus* sp. KR25; (**c**) *E. coli* KR29.

The growth curve analysis show that *Pseudomonas* sp. KR23, *Lysinibacillus* sp. KR25 and *E. coli* KR29 have acquired a certain level of tolerance to copper metals as shown by the high growth rate in the presence of this metal. The growth rate of these isolates in the presence of copper is more or less equal to the growth rate of the control. The growth rate of *Pseudomonas* sp. KR23 was unaffected by the presence of chromium. However the growth of *Lysinibacillus* sp. KR25 and *E. coli* KR29 was hindered in the presence of chromium (Figure 1).

### 2.2. Amplification of Heavy Metal Resistance Genes

**Figure 2:**
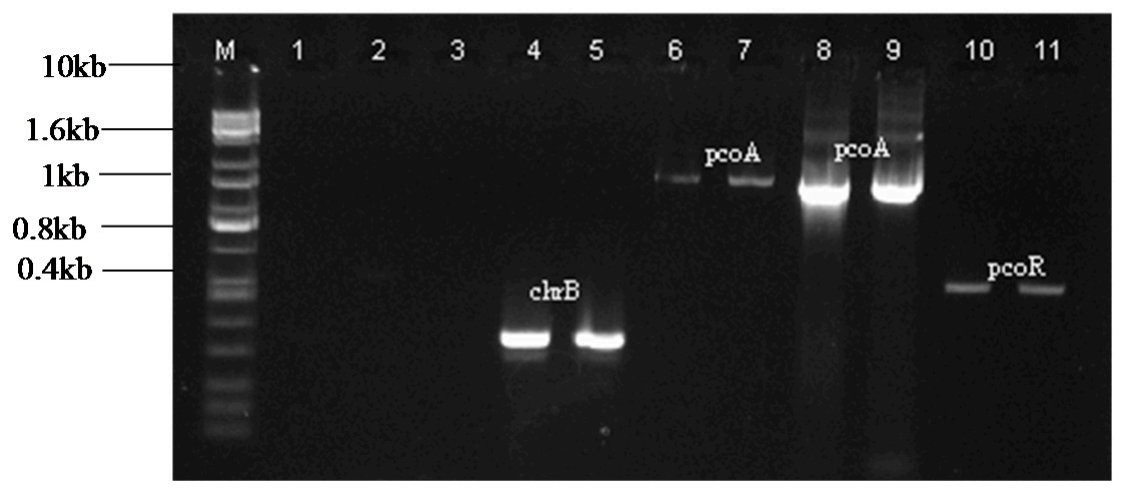
PCR amplification of chrB, pcoA and pcoR genes from the isolates *Pseudomonas* sp. KR23, *Lysinibacillus* sp. KR25 and *E. coli* KR29. The amplicons were visualized on a 1% agarose gel. Lanes represent the following: **M**. KAPA Universal Ladder; **1**. Negative control for chrB primers; **2**. Negative control for pcoA primers; **3**. Negative control for pcoR primers; **4**. chrB_23; **5**. chrB_23; **6**. pcoA_25; **7**. pcoA_25; **8**. pcoA_29; **9**. pcoA_29; **10**. pcoR_29 and **11**. pcoR_29.

The chrB primers produced a fragment of approximately 400 bp (Figure 2) in *Pseudomonas* sp. (KR23). This amplicon was designated chrB_23. Amplification with the pcoA primers produced a fragment of 1.7 kb in the bacterial strains *Lysinibacillus* sp. (KR25) and *E. coli* (KR29) (Figure 2). These amplicons were designated as pcoA_25 and pcoA_29, respectively. The pcoR primers produced a band of approximately 600 bp (Figure 2) in *E.coli* (KR29), which was designated pcoR_29.

### 2.3. Homology Analysis of Amplified Genes

The putative gene and protein sequences of chrB_23, pcoA_25, pcoA_29 and pcoR_29 were submitted in the GenBank under the accession numbers KP 745560-KP 745563, respectively.

**Figure 3:**
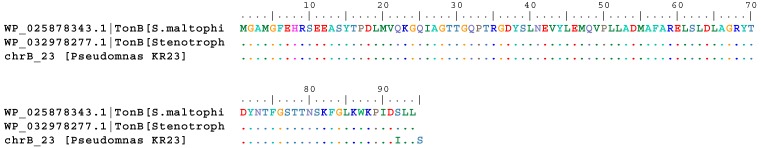
Alignment of the TonB protein from *S. maltophila* (WP033832814.1) and *Stenotrophomonas* sp. RIT309 (WP032978277.1) with the predicted chrB_23 protein.

The BLASTp analysis for chrB_23 aligned with 100 protein sequences in the database. No putative conserved domains were detected. Ninety-eight percent of the query sequence from the chrB_23, showed a 99% homology to a tonB dependent receptor in *Stenotrophomonas* sp. RIT309 (WP032978277.1) and a TonB dependent receptor protein found in *S. maltophila* (WP033832814.1) (Figure 3).

**Figure 4:**
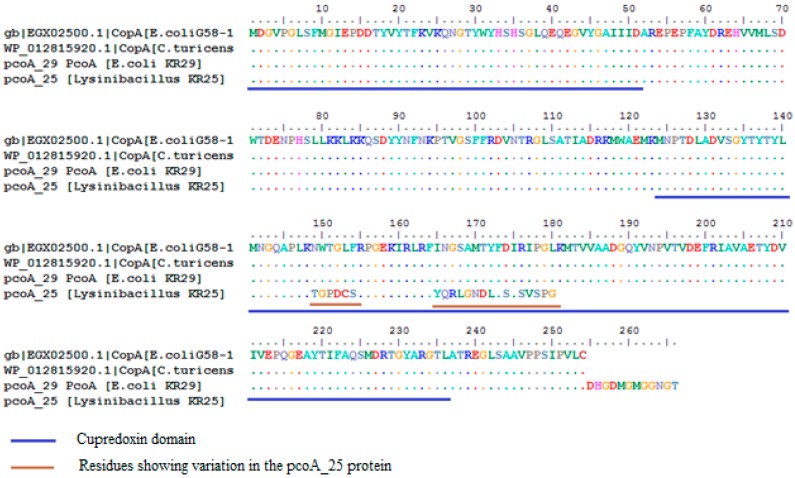
Alignment of the CopA protein from *E. coli* G58-1 (EGX02500.1) with the predicted partial PcoA proteins from *Lysinibacillus* KR25 and *E. coli* KR29.

**Figure 5:**
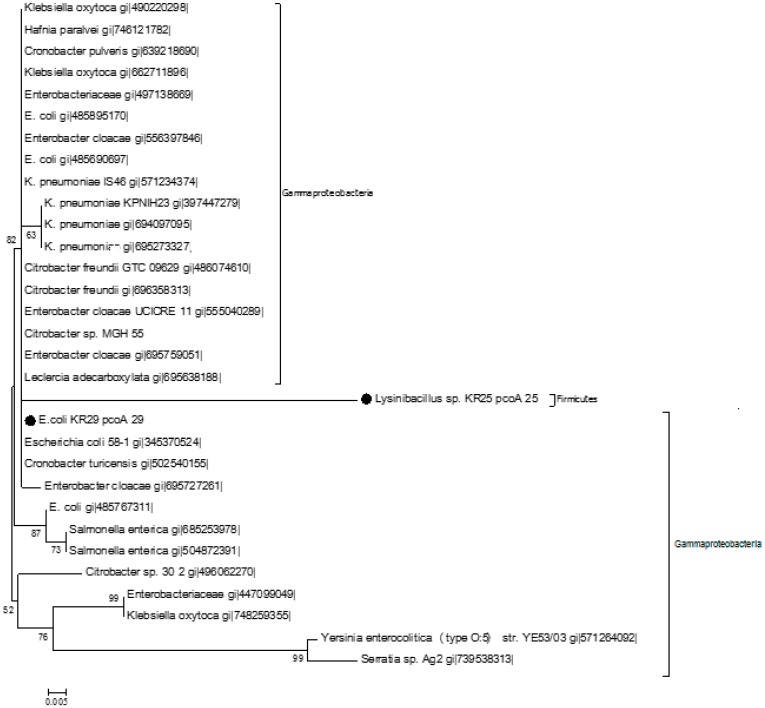
The evolutionary history was inferred using the Neighbor Joining Method [30]. The optimal tree with sum branch length 0.26187151 is shown. The percentage of replicate trees in which the associated taxa clustered together in the bootstrap test (1000 replicates) are shown next to the branches [31]. The evolutionary distances were computed using the *p*-distance method [32] and are in the units of the number of amino acid differences per site. The analysis involved 30 amino acid sequences. Evolutionary analysis was conducted using MEGA 6 [33]. The markers indicate the protein identified in this study.

Sequence comparisons of the pcoA fragment amplified from *Lysinibacillus* sp. KR25 (pcoA_25) showed high homology with copper resistance genes from other bacteria. For example, there was 90% homology with a copper resistant protein from both *Cronobacter turicensis* (WP012815920.1) and the copA protein in *E. coli* G58-1 (EGX02500.1) (Figure 4). Two putative conserved domains belonging to the *cupredoxin* domain of copper resistance protein family were detected from the Conserved Domain Database (Figure 4) [28]. The pcoA_25 protein was closely affiliated with orthologous copper resistance proteins from the following genera: *Klebsiella*, *Citrobacter*, *Escherichia*, *Cronobacter*, *Serratia*, *Achromobacter*, *Bordetella*, *Cellvibrio*, *Stenotrophomonas*, *Sulforovum*, *Pseudoxanthomonas*, *Burkhoholderia*, *Thiobacillus*, *Halomonas* and *Salmonella*. The orthologous sequences were inferred using OMA Browser [29]. Using the partial protein sequence of pcoA_29 as query on the BLASTp program, 100 sequences were retrieved. Ninety-five percent of the query showed 100% similarity to a copper resistance protein originating from *E. coli* G58-1 [EGX02500.1]. Ninety-five percent of the pcoA_29 query sequence also showed 100% similarity to a copper resistance protein copA found in *Cronobacter turicensis* (WP012815920.1). The sequences obtained from the *blastp* search were used to construct the phylogenetic tree (Figure 5).

**Figure 6:**
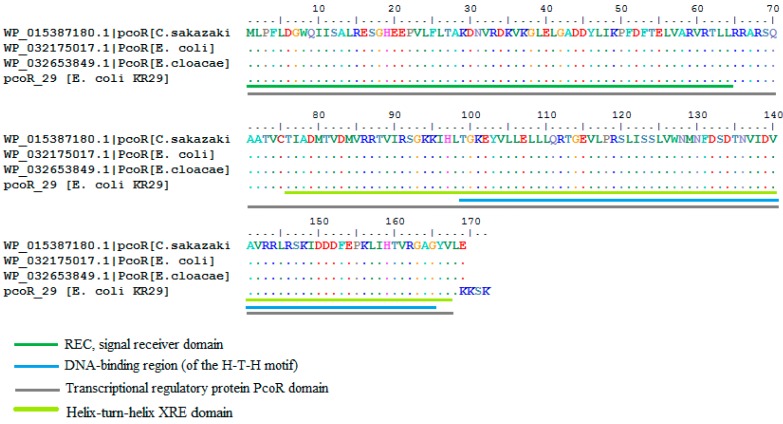
Alignment of the PcoR protein from *Cronobacter sakazakii* (WP015387180.1) with the predicted partial PcoR proteins from *Lysinibacillus* KR25 and *E. coli* KR29.

**Figure 7:**
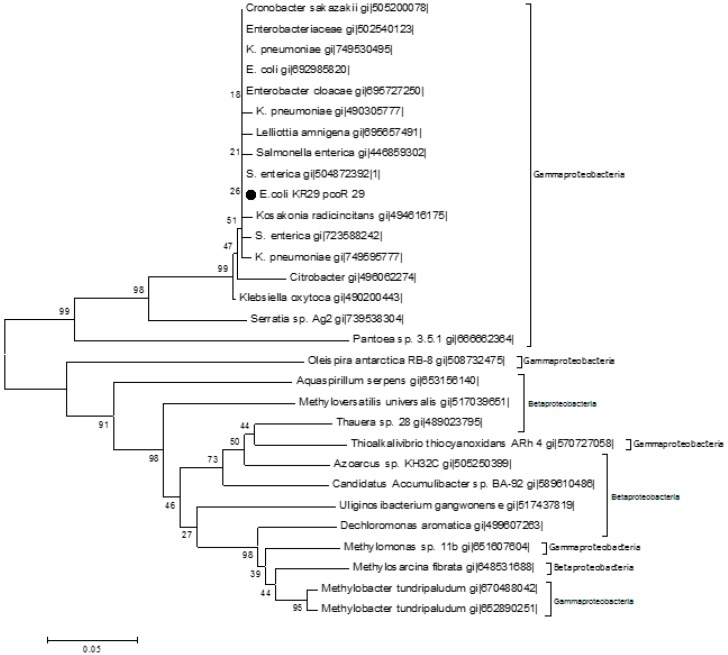
The evolutionary history was inferred using the Neighbor Joining Method [30]. The optimal tree with sum branch length 1.34233202 is shown. The percentage of replicate trees in which the associated taxa clustered together in the bootstrap test (1000 replicates) are shown next to the branches [31]. The evolutionary distances were computed using the *p*-distance method [32] and are in the units of the number of amino acid differences per site. The analysis involved 30 amino acid sequences. Evolutionary analysis was conducted using MEGA 6 [33]. The markers indicate the protein identified in this study.

The partial protein sequence pcoR_29, resulted in 100 hits using the BLASTp program. Ninety-eight percent of the query cover showed 99% similarity to a transcriptional regulatory protein pcoR found in *E. coli* (WP032175017.1), *Enterobacter cloacae* (WP032653849.1) and *Cronobacter sakazakii* (WP015387180.1) (Figure 6). Three major functional domains were identified namely; the REC signal receiver domain, the transcriptional regulatory protein PcoR domain and the Helix-turn-helix XRE domain (Figure 6). The pcoR_29 protein was closely affiliated with orthologous copper resistance proteins from the following genera: *Klebsiella*, *Escherichia*, *Cronobacter*, *Thiobacillus*, *Nitrosomonas*, *Methylophaga*, *Saccharophagus*, *Vibrio*, *Herminiimonas*, *Ralstonia*, *Pusillimonas*, *Hahella*, *Syemotrophomonas*, *Alteromonas*, *Pseudoalteromonas*, *Kangiella*, *Methylovorus*, *Dechloromonas* and *Thauera*. The orthologous sequences were inferred using OMA Browser [29] Selected sequences were used to construct a phylogenetic tree (Figure 7).

The partial primary structures of the proteins were used to predict their physico-chemical properties: the calculations were done using Expasy’s Prot Param Tool [34] (Table 1).

**Table 1:** Physicochemical properties of partial protein sequences.

Properties	ChrB_23	pcoA_25	pcoA_29	pcoR_29
Number of amino acids	95	180	266	172
Molecular weight (units)	10,558.9	20,544.1	29,571.6	19,565.8
Theoretical Pi	4.75	5.80	5.16	9.44
Total number of negatively charged residues (Asp + Glu)	12	23	33	23
Total number of negatively charged residues (Arg + Lys)	8	20	25	28
Extinction coefficient	12,950	34,380	45,840	15,470
Extinction coefficient *	-	34,380	45,840	15,470
Instability Index	12.158	25.06	20.63	41.56
Aliphatic index	74	63.89	70.38	109.30
Grand average of hydropathicity	−0.323	−0.612	−0.364	−0.103

The first extinction coefficient is based on the assumption that all cysteine residues appear as half cystines, and the second extinction coefficient * is based on assuming that no cysteine appears as half cysteine*.*

### 2.4. I-TASSER Structural and Functional Prediction Results

**Figure 8:**
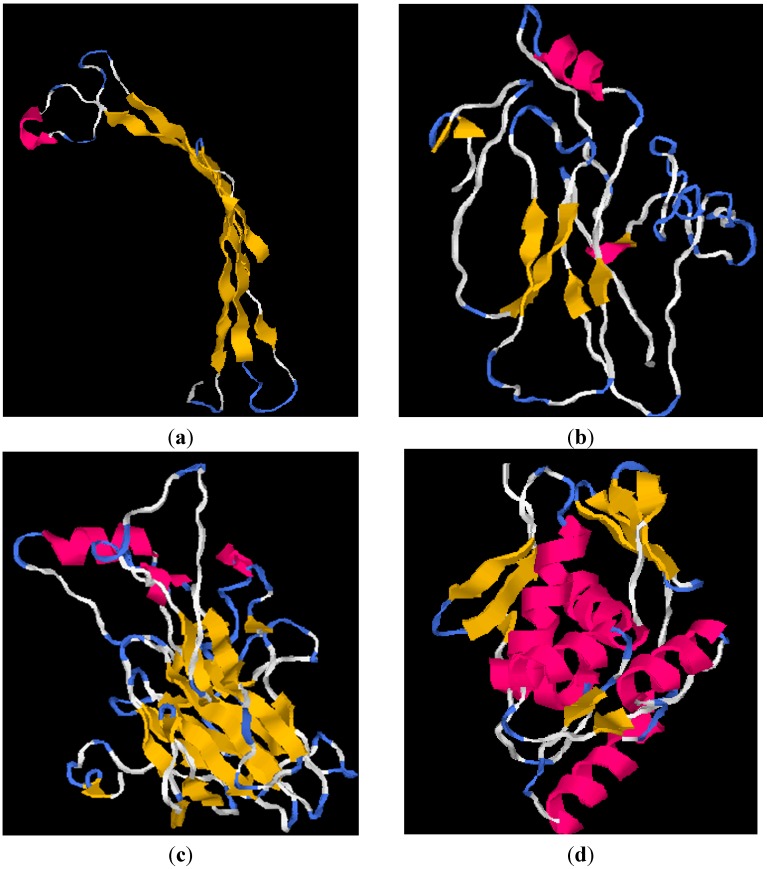
Shows the predicted structures of (**a**) chrB_23 (**b**) pcoA_25 (**c**) pcoA_29 and (**d**) pcoR_29 using I-TASSER. The models show the structure of the proteins using the following annotations: helices in magenta, sheets in yellow, turns in light blue and all the other residues in white.

The 3D-models of chrB_23, pcoA_25, pcoA_29 and pcoR_29 were constructed using I-TASSER are shown in Figure 8. Five models were constructed for each input sequence and the first model was selected according to the statistical analysis of predicted I-TASSER structures (Table 2). The results of the C-score, TM-score and RMSD calculated in I-TASSER are shown in Table 2. The biological functions of the proteins were predicted using the Cofactor program [28]. The result obtained for chrB_23 show that this protein might be responsible for siderophore transport with 51% probability. Iron siderophore transport involves low molecular weight Fe(III) chelating substances involved in the transportation of iron into and out of the cell. The partial protein pcoA_25 and pcoA_29 might be responsible for oxidation-reduction process with probabilities of 72% and 85%, respectively. PcoR_29 might be involved in phosphorelay signal transduction system with 94% probability. This transduction system involves autophosphorylation of a histidine kinase and the transfer of the phosphate group to an aspartate that then acts as a phospho-donor to response regulator systems [28].

**Table 2:** Statistical analysis of predicted I-TASSER structures.

Properties	chrB_23	pcoA_25	pcoA_29	pcoR_29
C-score	−1.34	−0.94	−0.02	1.20
TM-score	0.55 ± 0.15	0.60 ± 0.14	0.71 ± 0.12	0.88 ± 0.07
Root Mean Square Deviation (Å)	6.6 ± 4.0	7.1 ± 4.2	6.0 ± 3.7	2.7 ± 2.0

### 2.5. Ramachandran Plot Analysis

**Figure 9:**
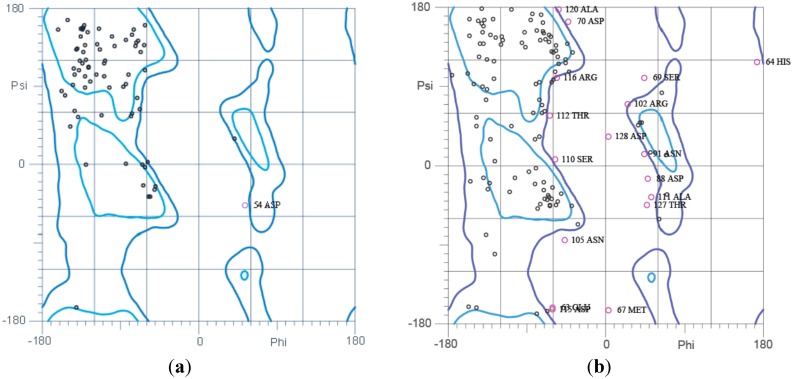

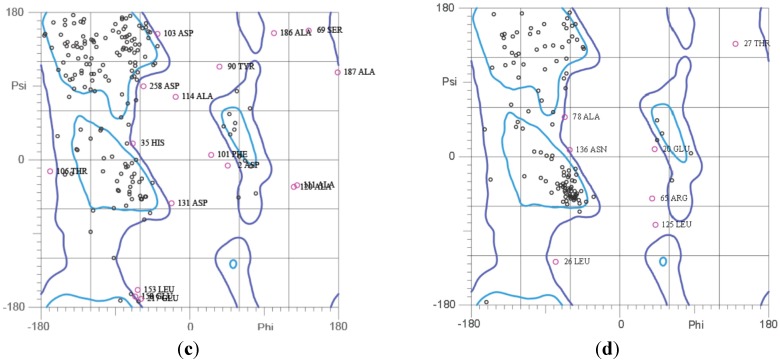
Ramachandran Plot Analysis (Ramachandran of I-TASSER MODEL structures predicted for (**a**) chrB_23 (**b**) pcoA_25 (**c**) pcoA_29 and (**d**) pcoR_29.

To investigate the accuracy and stereochemical quality of the predicted I-TASSER MODELS, the program MolProbity was used [35]. The results obtained from Ramachandran plot analsysis are shown in (Figure 9). The Ramachandran Plot for chrB_23 (Figure 9a) showed that 73.1% (68/93) of all the residues were in the most favored (98%) regions. About 95.7% (89/93) of the residues are in allowed (>99.8%) regions. There were four outliers (phi, psi) as shown by the purple markers; residue 28 Glycine (−64.4, 86.4), 31 Glycine (−66.7, −90.7), 54 Asparagine (53.2, −48.0) and 90 Isoleucine (67.2, 151.5).

The analysis for pcoA_25 (Figure 9b) shows that 60.1% (107/178) of the residues were in the most favored regions and 84.8% (151/178) of all the residues were in the allowed regions. A total of 27 outliers were noted.

Figure 9c indicates the Ramachandran plot for pcoA_29. Seventy-three percent (193/264) of residues were in the most favored regions. Approximately 93.2% (246/264) of residues are in the allowed regions. There were 18 outliers that were detected.

For the pcoR_29 protein translated from *E. coli* KR29 (Figure 9d), some of the residues 84.7% (144/170) appeared in most favored regions. About 95.3% (162/170) of the residues appeared in the allowed region. There were eight outliers.

### 2.6. Plasmid Profiling and Curing

**Figure 10:**
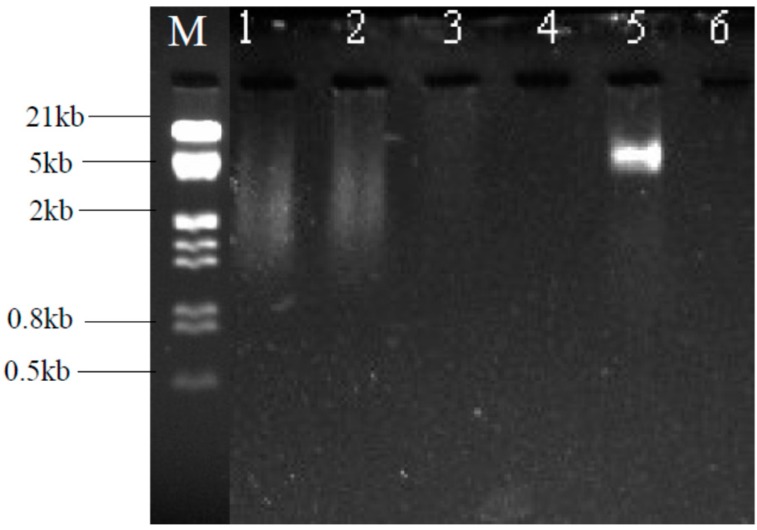
Plasmid profiles of wild strains of heavy metal resistant isolates and their cured derivatives. **M**. Lambda DNA + HindIII/EcoRI Marker; **1**. *Pseudomonas* sp (KR23) WT; **2**. *Pseudomonas* sp. (KR23) C; **3**. *Lyisnibacillus* sp. (KR25) WT; **4**. *Lysinibacillus* sp. (KR25) C; **5**. *E. coli* (KR29) WT; **6**. *E. coli* (KR29) C. WT = wild type strain; C = cured derivative.

The plasmid profiles of the three wild-type strains and their cured derivatives are shown in Figure 10. In this study plasmids could not be isolated from *Pseudomonas* KR23 and *Lysinibacillus* KR25 with the two methods despite repeated experimentation*.* A plasmid of approximately 5 kb was isolated from the wild type *E. coli* KR29. With curing the bacterium lost its plasmid.

Despite the curing of *Pseudomonas* sp. (KR23), it still maintained its antibiotic resistance to Streptomycin, Cephalothin acid, Vancomycin, Ampicillin and Amoxicillin and the strain still retained its heavy metal resistance against Cr and Cu (Table 3 and Table 4). After curing, *Lysinibacillus* KR25 lost its antibiotic resistance to Streptomycin and its resistance to copper. *Escherichia coli* KR29 did not register a drastic change in its antibiotic profile. However, the isolate lost its Amoxicillin resistance but still retained its heavy metal resistance (Table 4).

**Table 3:** Antibiotic profiles of isolates before curing [3].

Antibiotic	KR23	KR25	KR29
Streptomycin	NZ	NZ	16(S)
Cephalothin acid	NZ	27(S)	13(S)
Tobramycin	20(S)	18(S)	18(S)
Neomycin	20(S)	17(S)	13(S)
Tetracycline	23(S)	21(S)	19(S)
Cotrimoxazole	28(S)	22(S)	22(S)
Vancomycin	NZ	18(S)	NZ
Ampicillin	NZ	27(S)	14(S)
Amoxicillin	NZ	30(S)	12(R)

Letter in parenthesis indicates sensitivity: R—Resistant, S—Susceptible, NZ—no zone.

**Table 4:** Antibiotic and heavy metal resistance profiles of isolates after curing.

Antibiotic	KR23	KR25	KR29
Streptomycin	NZ	NG	18(S)
Cephalothin acid	NZ	NG	16(S)
Tobramycin	31(S)	NG	16(S)
Neomycin	15(S)	NG	15(S)
Tetracycline	30(S)	NG	23(S)
Cotrimoxazole	33(S)	NG	24(S)
Vancomycin	NZ	NG	NZ
Ampicillin	NZ	NG	17(S)
Amoxicillin	NZ	3G	15(S)
Heavy metal resistance profiles after curing			
Control	+++	+++	+++
Chromate (0.2mm)	++	NG	NG
Copper (0.6mm)	++	NG	++

Letter in parenthesis indicates sensitivity: S—Susceptible, NZ—no zone, NG—no growth; +++—exceptional growth; ++—average growth.

## 3. Discussion

### 3.1. Chromate Resistance Genes

*Pseudomonas* KR23 is resistant to chromate, and showed a minimum inhibitory concentration (MIC) value of 0.2 mM to chromium and grew exceptionally well in the presence of chromate (Figure 1) Therefore, it was suspected that this isolate may be harboring chromate resistance genes. The chrB gene is purported to play a regulatory role in the expression of the ChrA transporter [14] for the regulation of chromate resistance. The primers for the chrB gene amplified a single fragment of about 400 bp in *Pseudomonas* KR23 in this study. The fragment size is similar to the target gene in *C. metallidurans* CH34 [14]. However, after comparing the sequence with similar sequences from the NCBI database, it was found that the chrB_23 protein was *not* associated with chromate resistance. However, the chrB_23 protein sequence was closely related mainly to the TonB dependent receptor protein. Cofactor analysis infers that the chrB_23 gene amplified from *Pseudomonas* KR23 could possibly be a siderophore, which is involved in iron transport. Therefore the putative chromate resistance gene obtained in this study is not the targeted regulatory gene for chromate resistance.

### 3.2. Copper Resistance Genes

The main genetic determinants responsible for copper resistance in bacteria are the *pcoA* gene that encodes for a multi-copper oxidase (MCO) [10] and the *pcoR* gene that codes for the DNA binding repressor protein [6,7]. Out of the three isolates that were amplified with primers for the *pcoA* and pcoR genes, the pcoA gene was preset in two isolates, namely, *Lysinibacillus* KR25 [KJ935917] and *E. coli* KR29 [KJ935918] (Figure 1). In a previous study, these two isolates exhibited copper resistance and had (MIC) values of 1 mM and 0.6 mM, respectively [3]. The growth patterns of the three isolates in the presence of copper were more or less similar to the control (Figure 1). High concentrations of copper of up ±0.86 mg/L were detected in the Klip River [3]. Therefore, bacteria that are exposed to such high levels of heavy metals in the environment may have adapted to these stresses by developing several resistance or coping mechanisms [36]. The detection of the copA/PcoA genes in *Lysinibacillus* sp. KR25 and *E. coli* KR29 possibly mean that the copper efflux mechanism is operating in these organisms because the P-type ATPase CopA pumps excess copper out of the cytoplasm [37]. The *pcoA* (*copA*) genes encoding multicopper oxidases has been described in the following plasmids pPT23D, pRJ1004 and pMOL30 from the following microorganisms; *E. coli* RJ92, *Pseudomonas* *syringae pv.* *tomato* PT23 and *Cuprividus metallidurans*, respectively [38,39,40]. Genes identical to the copper-resistant genes, pcoR and pcoA from *E. coli*, were amplified by PCR from a 1.4-Mb megaplasmid detected in a root nodule bacterium*, Sinorhizobium meliloti* CCNWSX0020 [41].

The phylogenetic tree (Figure 5) shows that the pcoA_25 protein from *Lysinibacillus* KR25 and the pcoA_29 protein from *E. coli* KR29 are closely associated with copA proteins from *Klebsiella*, *Salmonella*, *Citrobacter*, *Enterobacter*, *Escheriachia*, *Serratia* and *Cronobacter* strains, respectively. All of the bacterial genera in Figure 5 are in the phylum *Gammaproteobacteria*. However, *Lysinibacillus* KR25 from which the query sequence (pcoA_25) was obtained belongs to the phylum *Firmicutes*. The phylogenetic tree (Figure 5) infers that pcoA_25 is a paralogous gene, since it seems like it has received a mutation that gives rise to a new gene.

The PcoA (Figure 5) and PcoR (Figure 7) orthologs from the same phylum were split into different clusters. The *Proteobacteria* orthologs in particular clustered in different positions, clearly inferring horizontal gene transfer events.

The putative conserved domains detected in pcoA_25 and pcoA_29 are part of the *cupredoxin* domain of the copA copper resistance family of genes. CopA is related to the enzymes laccase and l-ascorbate oxidase, both of which are copper containing enzymes. It forms part of the regulatory protein *Cue* operon, which utilizes a cytosolic metalloregulatory protein CueR that induces the expression of *copA* and CueO in the presence of high concentrations of copper. Structurally, the cupredoxin-like fold comprises of a beta-sandwich with seven strands in two beta-sheets, which are usually arranged in a Greek-key beta-barrel. A multicopper oxidase CueO contains three repeats as shown by the I-TASSER models for pcoA_25 and pcoA_29 [28].

The partial protein sequence pcoR_29, resulted in 100 hits using the BLASTp and showed 99% similarity to a transcriptional regulatory protein pcoR found in *E. coli* (WP032175017.1), *Enterobacter cloacae* (WP032653849.1) and *Cronobacter sakazakii* (WP015387180.1). Cofactor analysis elucidated that this protein may be involved might be involved in phosphorelay signal transduction system with 94% probability. The presence of the REC domain in pcoR_29, makes this protein suitable for this function because this domain contains a phosphoacceptor site that is phosphorylated by histidine kinase homologs; usually found at the *N*-terminal of a DNA binding effector domain. The Helix-turn-helix XRE-domain was also present in the pcoR_29 protein. This domain belongs to the xenobiotic response element family of transcriptional regulators.

### 3.3. Plasmid Isolation and Curing

Most of the studies involving copper resistance in bacteria have shown that the *pco* system is well characterized in *E. coli* strains where the genes are located in plasmids [42,43]. In this study, it appears that in *E. coli* KR29, the pcoA and pcoR genes are located on the chromosome since the isolate still retained its antibiotic and copper resistance after the plasmids were successfully eliminated (Figure 7).

This study showed that the *pcoA* gene was present in *Lysinibacillus* sp. After curing, the isolate lost both its antibiotic and copper resistance properties (Table 3 and Table 4). The strain lost its antibiotic resistance against Streptomycin, therefore it can be elucidated that the resistance determinant for this drug is present on a plasmid. However, following plasmid isolation no DNA band/s were observed on ethidium bromide stained gels. It has been noted that plasmid isolation can be difficult in some bacterial strains [44].

Environmental bacteria have been shown to be a great source of heavy metal resistance genes and a potential source of novel heavy metal genes. Bacteria can acquire genes for heavy metal and antibiotic resistance in several ways, such as horizontal gene transfer, which could arise from the presence of genetic mobile elements in bacteria, such as plasmids, transposons, integrons, genomic islands and bacteriophages [45]. The occurrence of the copper resistance gene among the isolates in this study is most probably due to one or may of these mechanisms.

Antibiotic resistance may be due to enzyme inactivation, lack of target sites for drugs, intracellular drug accrual, or the presence of genes that enable microorganisms to acquire antibiotic resistance [46]. Antibiotic resistance genes tend to reside either on the plasmid [47] or on the chromosome of the bacteria [48]. In this study, the strain *Pseudomonas* KR23 retained its antibiotic resistance to Streptomycin, Cephalothin acid, Vancomycin, Ampicillin and Amoxicillin after curing. Since plasmid isolation was unsuccessful for this strain it is difficult to deduce whether the resistance determinants are present on the plasmid or on the chromosome.

### 3.4. Physico-Chemical Properties

The predicted physico-chemical properties of protein sequences can give valuable insights to the characteristics of protein. For instance, protein with an instability index smaller than 40 are considered to be stable while those with an instability factor above 40 are predicted to be unstable [49]. In this study the ProtParam Tool was used to characterize the predicted physicochemical properties of the partial protein sequences that were obtained in this study (Table 1). The pcoA proteins from *Lysinibacillus* KR25 and *E. coli* KR29, the chrB protein from *Pseudomonas* sp. KR23 can be considered stable, since their instability indices were 25.06, 20.63 and 12.16, respectively. The high instability index for pcoR_29 from *E. coli* KR29 predicts that the protein is unstable. The high extinction coefficient of pcoA_29 indicates the presence of high concentrations of Cysteine (Cys), Tryptophan (Trp) and Tyrosine (Tyr) residues, while the low extinction coefficient shown by the chrB_23 indicates low concentrations of these amino acids in the proteins. The high aliphatic indices shown by the four partial proteins are indicators that the proteins may be stable over a wide temperature range [49]. The Grand Average of Hydropathicity Value (GRAVY) predicts the hydrophobic and hydrophilic nature of the protein. A negative value indicates that the protein is hydrophilic and a positive value indicates that the protein could be hydrophobic [50]. The GRAVY values for all four proteins are negative suggesting that they are most likely hydrophilic.

### 3.5. Protein Structures

The tool that was used in this study to predict the protein structures of the partial proteins was I-TASSER, since it was reported to reliably predict partial structures of a source protein [23]. I-TASSER initiates the structure prediction process by performing threading to identify template Protein Database Structures that are similar to the query sequence. Structural fragments that are cut from the chosen templates are then used to construct the protein models [23]. The C-score output of I-TASSER was used to distinguish the foldable and non-foldable sequences because it is founded on the probability that the sequence is homologous to known structures at the subsequence level rather than globally. Structure predictions with a TM score greater than 0.6 was defined as “good”, “decent” (TM-score between 0.3 and 0.6) or “bad” (TM-scores less than 0.3) [23].

The structure predicted for chrB_23 was decent taking into account its high TM-score of 0.55. This structure is most likely to fold as depicted by the moderately high C-score value of −1.35. The Ramachandran plot for chrB_23 confirms the good stereo-chemical structure of chrB_23, since 95.7% of all the residues lie in the allowed regions and only six outliers were noted.

The C-score (−0.94) obtained for the pcoA_25 protein structure suggests that the partial protein is most likely to fold into a stable structure that is considered to be of good quality as shown by the high TM-score of 0.66. The Ramachandran Plot for pcoA_25 shows that the structure is of reasonable quality with 84.8% of the residues lying in the allowed region.

The structure predicted for pcoA_29, is most likely to fold considering the high C-score value of −0.02 and it is considered to be “good” considering the TM-value of 0.60 and the Ramachandran plot.

For pcoR_29, the predicted structure is most likely to fold into a very stable structure because its C-score value was very high 1.20 and the quality of the prediction is very good, considering the high TM-score of 0.88 and the high quality of the structure was confirmed by the Ramachandran Plot, which shows 95.3% of the residues lying in the allowed region. Computational methods aid in the prediction of protein structure rapidly and economically [49]. The results of this analysis indicated that the predicted structures are ready to be verified *in vitro* and will enable further research to be carried out on heavy metal resistance proteins. I-TASSER is a superior computational method compared to other protein prediction program, since it takes into account the full length of the sequence and it uses several templates in the PDB database to construct the full structure. Through the threading method used by I-TASSER complete structures were constructed and predicting the foldability of a protein the C-score analysis is accurate since it considers the subsequence level [23].

## 4. Experimental Section

### 4.1. Growth Studies

Growth studies of the bacterial isolates was carried out in 250 mL flasks containing 50 mL LB medium supplemented with 0.2 mM concentration of CdCl_2_, PbCl_2_, ZnCl_2_, FeSO_4_·7H_2_O, NiCl_2_·6H_2_O and K_2_Cr_2_O_7_. Flasks were inoculated with 0.5 mL of overnight culture and incubated on a rotatory shaker (150 rev/min) at 30 °C. The optical density was measured every four hours using a UV-spectrophotometer at 600 nm (Nanocolour UV/VIS Spectrophotometer, Mahery-Nagel, Düren, Germany) [36].

### 4.2. Genomic DNA Extraction

Genomic DNA was extracted by using the ZR Fungal/Bacterial DNA Extraction Kit (Zymo Research, Irvine, CA, USA) according to the manufacturer’s protocol. DNA was extracted from one chromate and two copper resistant bacterial strains (Table 5) isolated from the Klip River, Johannesburg, South Africa The quantity and quality of the DNA was assessed by reading its absorbance at 260 and 280 nm using Nanodrop 2000c Spectrophotometer (Thermofischer Scientific, Wilmington, DE, USA) [51]. The DNA samples were also electrophoresed on a 1.0% (*w*/*v*) agarose gel and stained with ethidium bromide to confirm its quality.

**Table 5:** Bacterial strains used for heavy metal gene amplification.

Strain	Source	Location	Accession Number
*Pseudomonas KR23*	Klip River (water sample)	South Africa	KJ935916
*Lysinibacillus KR25*	Klip River (water sample)	South Africa	KJ935917
*Escherichia coli*	Klip River (water sample)	South Africa	KJ935918

### 4.3. Amplification of Heavy Metal Resistant Genes

The following heavy metal resistance genes (*pcoR*, *pcoA*, and *chrB*) were amplified using specific primers (Table 6). PCR was performed in 50 µL reaction mixture containing 21 µL of 2x PCR Mastermix Emerald Amp R MAX HS Master Mix, (Takara, Kyoto, Japan), 1 µL DNA template, 1 µL of each forward and reverse primer (0.2 µM), and 22 µL of PCR quality water. The PCR amplification of the target DNA was carried out in a T100™ Bio-Rad Thermocycler (Bio-Rad, Hercules, CA, USA). The following thermal cycling parameters were used: initial denaturation step at 95 °C for 5 min, followed by 35 cycles at 94 °C for 90 s (denaturation), 57 °C for 90 s (annealing), and 72 °C for 2 min (elongation). This was followed by a final extension step of 72 °C for 7 min. A cooling temperature of 4 °C was applied [6,14].

**Table 6:** Heavy metal resistance gene primers used in this study.

Gene to Be Amplified	Primer Sequences	Orientation	Corresponding Microbe with Heavy Metal Resistance Gene	Exact Length of Amplified Region
*chrB*	GTCGTTAGCTTGCCAACATC	Forward	*C. metallidurans* CH34	450 [14]
	CGGAAAGCAAGATGTCGATCG	Reverse		
*pcoA*	CGTCTCGACGAACTTTCCTG	Forward	*E. coli* E8739	1791 [6]
	GGACTTCACGAAACATTCCC	Reverse	Plasmid pPA87	
*pcoR*	CAGGTCGTTACCTGCAGCAG	Forward	*E. coli* E8739	636 [6]
	CTCTGATCTCCAGGACATATC	Reverse	Plasmid pPA87	

### 4.4. Gel Electrophoresis

The amplicons were electrophoresed in a Bio-Rad electrophoresis system for 1 hour at 90 V in 1× TBE buffer. The images of the gels were captured in a Bio-Rad Gel Doc™ EZ Imager (Bio-Rad, Hercules, CA, USA) using ImageLab™ Software version 5.0 (Bio-Rad, Hercules, CA, USA). Each gel contained 5 µL of KAPA Universal Ladder (KAPA Biosystems, Boston, MA, USA) in the first well. The image was analyzed using the ImageLab™ Software to determine the size of the bands produced in each lane.

### 4.5. PCR Product Purification and Sequencing of pcoA, pcoR and chrB Genes

The PCR products were purified using the GeneJet™ PCR Purification Kit (Fermentas, Hanover, Germany) according to the manufacturer’s protocol. The purified DNA was stored at −20 °C and later sequenced using the Sanger method [52] by Inqaba Biotechnologies Industry, Pretoria, South Africa.

### 4.6. Homology Analysis of Amplified Genes

The partial gene sequences obtained in this study (*pcoA*, *pcoR* and *chrB* genes) were translated using the Expasy Translate Tool. The longest open reading frames (ORFs) were selected from the initiator (the first methionine (ATG) at the beginning of the sequence up to a stop codon (TAA, TAG and TGA) [34]. The protein sequences were then aligned and compared with other proteins in the Genbank by using the NCBI Basic Local Alignment Search Tools (BLASTp) program using the default settings; (Word size: 3, Expect value: 10, Gapcosts: 11.1, Matrix: BLOSUM62, Filter string: F, Window size: 40, Threshold: 11, Composition based stats: 2 [53]. Based on the scoring index, the most similar sequences were aligned with other heavy metal resistance proteins using MAFFT Multiple Sequence Alignment Software Version 7 [54]. Phylogenetic analysis and similarity index was generated using the program MEGA 6 [33] and compared with other known species. The heavy metal resistance gene sequences of the bacteria have been deposited in Genbank. The physico-chemical properties of the partial protein sequences were predicted using the Expasy’s Prot Param Tool [34] and the 3D structures of the partial proteins were constructed I-TASSER [22]. Function prediction analysis was carried out by Cofactor [28]. The modeled structure was then analyzed using Molprobity [35]. This program checks the stereochemical quality of a protein structure, producing a number of PostScript plots assessing its overall and residue-by-residue geometry.

### 4.7. Isolation of Plasmid DNA

Plasmid DNA was isolated from the isolates, according to the Alkaline Lysis method [55].

### 4.8. Plasmid Curing

To determine if the resistance gene is encoded by a plasmid, ethidium bromide was used to eliminate the plasmids from the strains and heat treatment was applied as a control. The strains were grown in the presence of ethidium bromide (100 µg/mL) and then spread on Nutrient Agar (NA) plates each containing the metals, Zn, Pb, Cr, Cd and Cu. The control plates did not contain the heavy metals. The experiment was replicated and the plates were incubated at 30 °C. Plasmids were considered to be eliminated from those bacterial strains that grew on the metal free medium [56]. For heat treatment, the strains were grown at 45 °C and sub-cultured into fresh medium. The culture was plated onto NA containing its respective metals and its metal-free form [57].

### 4.9. Antibiotic Profiles of Wild Type Strains and Cured Derivatives

Antibiotic profiles of wild type strains and cured derivatives were constructed using the Kirby Bauer method [58]. Overnight grown cultures of wild type bacterial strains and cured derivatives (100 µL) were transferred to Muller-Hinton agar plates and spread over the surface of the medium with a sterile swab. After 10–15 min, antibiotic discs were placed on the medium and then incubated at 37 °C for 24 h. The zone of inhibition was measured in millimeters (mm). Bacterial strains were considered to be susceptible when the inhibition zone was more than 12 mm in diameter [36]. Tests were performed in triplicate. The following antibiotics discs were used: Ampicillin (10 µg/mL), Amoxcyllin (10 µg/mL), Cephalothin acid (30 µg/mL), Cotrimoxazole (25 µg/mL), Neomycin (30 µg/mL), Streptomycin (10 µg/mL), Tetracycline (30 µg/mL), Tobramycin (10 µg/mL), and Vancomycin (30 µg/mL).

## 5. Conclusions

The heavy metal resistances of the isolates *Lysinibacillus* sp. KR25, *E. coli* KR29 and *Pseudomonas* sp. KR23 were confirmed using different methods. A detailed analysis conformed that the two isolates namely, *Lysinibacillus* sp. KR25 and *E. coli* KR29 possess *PcoA* genes and *E. coli* KR29 has a *PcoR* determinant. These genes are most likely responsible for their copper resistances. The putative chrB determinant obtained from *Pseudomonas* sp. KR23 is not involved in chromate resistance. The PcoA and PcoR genes obtained in this study have been predicted to form stable structures, therefore, these genes may be used in biotechnological applications, such as the construction of biosensors, whereby these isolated genes may be combined with reporter genes, such as *gfp*, *lacz* and *lux* genes to detect the contaminants in the environment.
